# Effect of urine storage conditions on circulating anodic antigen detection using the UCP-LF CAA test

**DOI:** 10.3389/fpara.2026.1894516

**Published:** 2026-10-05

**Authors:** Pytsje T. Hoekstra, Govert J. van Dam, Maureen Blokland-Flink, Danielle de Jong, Said M. Ali, Mohammed N. Ali, Naomi C. Ndum, Stefanie Knopp, Paul L.A.M. Corstjens, Lisette van Lieshout

**Affiliations:** 1 Leiden University Center for Infectious Diseases (LUCID), Leiden University Medical Center, Leiden, Netherlands; 2 Department of Cell and Chemical Biology, Leiden University Medical Center, Leiden, Netherlands; 3 Public Health Laboratory - Ivo de Carneri, Pemba, Tanzania; 4 Swiss Tropical and Public Health Institute, Allschwil, Switzerland; 5 University of Basel, Basel, Switzerland

**Keywords:** antigen detection, CAA, diagnostics, schistosomiasis, stability, UCP-LF CAA, urine storage

## Abstract

**Introduction:**

Schistosomiasis is a parasitic neglected tropical disease that remains a major public health problem in many African countries. Accurate diagnosis is essential for control, surveillance and elimination efforts. Conventional parasitological methods have limited sensitivity, particularly in low-intensity infections commonly observed after treatment or in low-endemic settings. Circulating anodic antigen (CAA), released by live *Schistosoma* worms, is a highly sensitive and specific marker of active infection. The up-converting reporter particle lateral flow (UCP-LF) CAA test enables non-invasive urine-based diagnosis and is increasingly applied in low-endemic and near-elimination settings. For operational feasibility, urine samples may need to be stored before testing, but the effect of different storage conditions on CAA detection has not been systematically evaluated.

**Methods:**

We investigated the effect of freezing and non-frozen storage on urine CAA detection using the UCP-LF CAA test, compared with freshly tested urine. The study was conducted among school-aged children in a near-elimination *Schistosoma haematobium* setting on Pemba, Tanzania. A total of 272 urine samples were tested fresh and after freezing at -20 °C for 5-7 days. For a subset of 153 samples with sufficient urine available, an additional aliquot was stored under non-frozen conditions (4-25 °C) and tested approximately 5 weeks after collection.

**Results:**

CAA positivity decreased from 44/272 (16.2%) in fresh urine to 35/272 (12.9%) after freezing. In the subset, positivity was 20/153 (13.1%) in fresh, 16/153 (10.5%) in frozen, and 24/153 (15.7%) in non-frozen stored urine. CAA concentrations showed strong correlation between storage conditions (Spearman’s ρ = 0.86–0.93, p<0.0001). Freezing was associated with a small but significant reduction in CAA positivity (p=0.004), while CAA concentrations showed a non-significant trend towards lower values after freezing. Non-frozen stored samples showed generally comparable results to fresh urine.

**Discussion/Conclusion:**

In conclusion, these findings suggest that flexible urine storage strategies may be feasible for delayed centralized CAA testing, although freezing may result in loss of detection among samples with CAA concentrations close to the assay cut-off.

## Introduction

Schistosomiasis is a parasitic disease caused by blood flukes of the genus *Schistosoma*, affecting over 200 million people worldwide, particularly in sub-Saharan Africa (Buonfrate et al., 2025). Transmission occurs through contact with freshwater contaminated with larval stages released by infected intermediate host snails. Accurate diagnosis of schistosomiasis is essential for effective control, surveillance, and elimination efforts. Conventional parasitological methods, such as urine filtration and stool microscopy, have limited sensitivity, particularly in low-endemic settings as well as after praziquantel treatment, when infection intensities are often low and egg excretion is intermittent (Hoekstra et al., 2021a). Detection of circulating anodic antigen (CAA), released by live *Schistosoma* worms, offers a sensitive measure of active infection (Corstjens et al., 2020). The up-converting reporter particle lateral flow (UCP-LF) CAA test has demonstrated high specificity and sensitivity for the detection of all *Schistosoma* species including *Schistosoma* hybrids and veterinary species (Knopp et al., 2015; van Dam et al., 2015a; van Dam et al., 2015b; Vonghachack et al., 2017; Clements et al., 2018; Sousa et al., 2019; Corstjens et al., 2020; Assaré et al., 2021; Hoekstra et al., 2021b) and complements or surpasses traditional egg-detection methods (Knopp et al., 2015; Clements et al., 2018; Assaré et al., 2021; Lorenz et al., 2025). CAA detection is increasingly applied across different settings, including low-endemic and near-elimination areas (Clements et al., 2018; Sousa et al., 2019; Corstjens et al., 2020; Assaré et al., 2021), post-treatment follow-up and pharmacokinetic and pharmacodynamic studies (Bustinduy et al., 2016; Sousa et al., 2019; Hoekstra et al., 2022a; Bustinduy et al., 2025; de Freitas Galvão et al., 2025), surveillance and sample pooling approaches (Corstjens et al., 2017; Hoekstra et al., 2020; Hoekstra et al., 2022c), non-endemic and migrant populations (Camprubí-Ferrer et al., 2021; Hoekstra et al., 2021b; Tamarozzi et al., 2021; Hoekstra et al., 2022b), and controlled human infection models (Langenberg et al., 2020; Abaasa et al., 2023; Koopman et al., 2023).

The detection of CAA in urine enables non-invasive testing using the UCP-LF CAA test, which can be performed in laboratories with basic equipment, making the test suitable for use in endemic settings (Downs et al., 2012; van Dam et al., 2013; Knopp et al., 2015; Gerstenberg et al., 2024; Bustinduy et al., 2025; Lorenz et al., 2025). For surveillance and research applications, urine samples may be collected in decentralized settings and transported to a central laboratory for subsequent testing. Storage prior to testing can facilitate centralized and retrospective analysis, potentially improving workflow efficiency and data comparability. CAA is considered stable because of its carbohydrate structure, which makes it resistant to degradation under a range of storage conditions, including prolonged storage room temperature, refrigeration, and freezing. This long-term stability of CAA is illustrated by its detection in Egyptian mummies, reflecting its preservation over thousands of years (Deelder et al., 1990; Miller et al., 1992). However, the effect of different urine storage conditions on measurable CAA concentrations has not been systematically evaluated under operational conditions. Here, we evaluated the effect of freezing for 5–7 days at −20 °C and non-frozen storage for approximately 5 weeks at 4-25 °C on CAA detection and concentrations, compared with freshly tested urine, using the UCP-LF CAA test.

## Methods

Urine samples were collected as part of a prospective diagnostic evaluation study performed among school-aged children in two schools on Pemba Island, United Republic of Tanzania, in February 2025. The main study evaluated the performance of a range of methods for *Schistosoma haematobium* infection diagnosis in a near-elimination setting (Ndum et al., 2026). CAA testing was prospectively included in the study using both freshly collected urine, tested on the day of collection, and frozen urine, tested after 5–7 days, reflecting the conventional approach used for CAA testing. Both analyses were performed at the Public Health Laboratory-Ivo de Carneri (PHL-IdC) on Pemba. During the study, we additionally introduced a third sample condition to assess a more practical field-to-laboratory scenario: approximately halfway through the study, when sufficient urine volume was available, a third aliquot was retained for non-frozen storage and subsequent transport to the Netherlands for CAA testing. This condition was included because in field settings where freezing is not available, urine may be stored refrigerated before transport. The resulting sample set was therefore smaller than that available for the prospectively planned fresh and frozen conditions. The study population and epidemiological setting have been described previously (Ndum et al., ). Ethical approval and participant consent procedures followed those described in the main study (Ndum et al., ).

The primary objective of the present sub-study was to compare CAA detection in freshly collected and frozen urine samples. This comparison was prospectively planned and performed for all urine samples with sufficient volume available (N = 272). The additional non-frozen condition was assessed in a subset with sufficient remaining urine volume (N = 153).

Urine samples were processed as follows: (1) in the first week, a first 0.5 mL aliquot was tested within 4 hours after collection (fresh) at PHL-IdC in Pemba; (2) a second 0.5 mL aliquot was frozen within 4 hours after collection, stored at −20 °C for 5–7 days, subsequently thawed, and tested at PHL-IdC during the second week; (3) a third 0.5 mL aliquot was stored at 4 °C, transported to the Netherlands under cooled conditions using ice packs at the end of the second week, and tested at the Leiden University Medical Center (LUMC) within 2–3 weeks of arrival. Thus, the third aliquot was tested approximately 5 weeks after sample collection (**Figure 1**).

**Figure 1:**
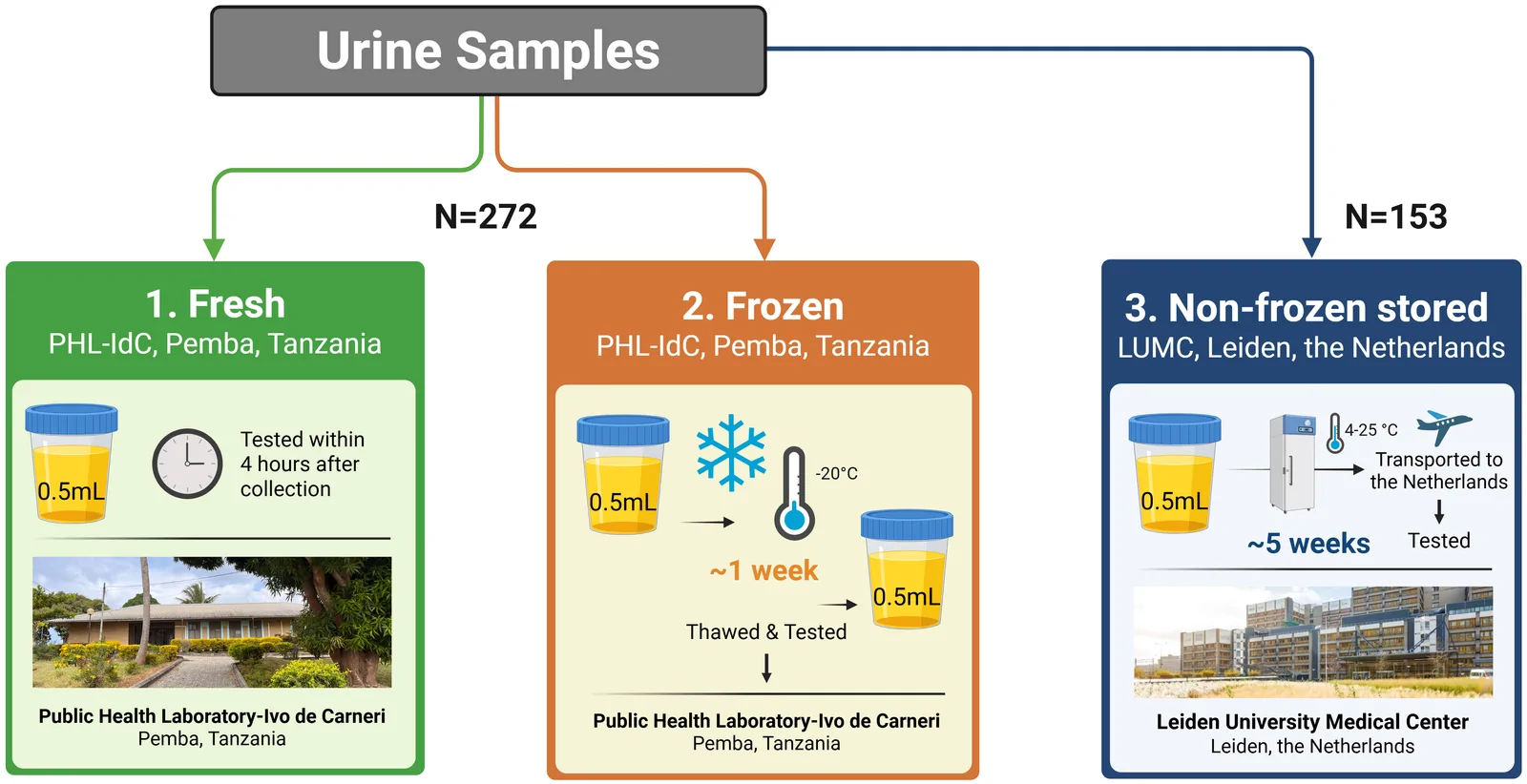
Schematic overview of urine sample processing and testing conditions (created with BioRender.com).

UCP-LF CAA testing followed standard procedures as described previously, using the UCAA*hT*417 dry format (Corstjens et al., 2020), which was selected based on a combination of high analytical sensitivity and suitability for execution in the field. All samples were analyzed using the same assay format, as well as the same batch of reagents, calibrators and controls and the same UCP-reader; fresh and frozen samples were analyzed on Pemba Island, whereas non-frozen stored samples were transported to and analyzed at LUMC, Leiden, the Netherlands. Briefly, samples were pre-treated with 12% trichloroacetic acid (TCA) to release CAA and precipitate proteins. Subsequently, 0.5 mL of the clear supernatant was concentrated using Millipore Amicon centrifugal filtration devices (Merck Chemicals BV, Amsterdam, the Netherlands) with a 10-kDa filter. Finally, the concentrated sample (20uL) was mixed with 100 μL assay buffer containing 50 ng of the highly sensitive reporter label (UCP; NaYF_4_:Yb^3+^,Er3+, 110x80 nm prisms; Intelligent Material Solutions, Princeton, NJ, USA). UCP materials were conjugated to anti-CAA antibodies and provided as freeze dried pellets (LyoSpheres™, batch #230718_540, BIOLYPH, Chaska, MN, USA). Lateral flow strips, comprising a test line with anti-CAA antibodies (batch #1745, MaximBio, Rockville, MD, USA), were added to the wells and immunochromatography was allowed for at least 3 hours. After incubation, bound CAA-UCP complexes were detected by scanning the LF strips with an ESEQuant reader (LR3, DIALUNOX, USA) adapted for the UCP-label (van Dam et al., 2013). In each run, a serial dilution series of a TCA-treated crude adult worm antigen (AWA-TCA) preparation spiked in CAA-negative urine was included to validate the cut-off of the test (2 pg/ml for the UCAA*hT*417 dry format) as well as to quantify the CAA concentration in the unknown samples. Samples with a CAA concentration ≥2 pg/ml were considered as positive (Corstjens et al., 2020). For visualization purposes, samples of which the CAA concentration was undetectable or below the cut-off of 2 pg/ml were assigned a fixed value equal to half the cut-off (1 pg/ml).

Data were analyzed using descriptive statistics. For qualitative outcomes, agreement between sample conditions was assessed using Cohen’s kappa coefficient, and differences in paired proportions were evaluated using the exact McNemar’s test. All paired samples were included in this analysis. For quantitative CAA concentrations, differences between paired measurements were assessed using the Wilcoxon signed-rank test, including all paired samples. Associations between paired measurements were assessed using Spearman’s rank correlation coefficient, and agreement and potential bias using Bland-Altman analysis of log-transformed concentrations. These analyses were restricted to samples with CAA concentrations ≥2 pg/ml under both conditions being compared. Mean and median CAA concentrations were likewise calculated only for samples with CAA concentrations ≥2 pg/ml under both conditions compared. Samples with CAA concentrations < 2pg/ml were considered negative for qualitative analyses. All tests were two-tailed, and p-values <0.05 were considered statistically significant. All analyses were performed using SPSS (version 29) and figures were generated using Graphpad Prism (version 10.6.1).

## Results

A total of 272 urine samples were tested both fresh and after freezing using the UCP-LF CAA test (**Tables 1**, **2**). The proportional Venn-diagram showed substantial overlap in CAA-positive samples between the two conditions (**Figure 2A**). Among fresh CAA-positive samples, 9 low-positive samples with CAA concentrations close to the positivity cut-off (2–5 pg/ml) tested negative after freezing, while no fresh CAA-negative samples tested positive after freezing (**Figure 3**; **Supplementary Table 1**). The difference in discordant pairs was statistically significant (McNemar’s test, p=0.004; **Table 2**). CAA concentrations tended to be lower after freezing, although this did not reach statistical significance (Wilcoxon signed-rank test, p=0.051; **Table 2**). A strong correlation was observed between quantitative CAA concentrations in fresh and frozen CAA-positive samples (N = 35, Spearman’s *ρ*= 0.93, *p* < 0.0001; **Table 2**; **Figure 3**). There was no statistically significant systematic bias in CAA concentrations between fresh and frozen samples. Fresh samples tended to have higher CAA concentrations compared to frozen samples (mean ratio 1.18, 95% limits of agreement 0.41-3.44, **Table 2**).

**Table 1:** UCP-LF CAA results from urine samples tested under different conditions.

	Full dataset (N = 272)		Sub-set (N = 153)		
	Fresh	Frozen	Fresh	Frozen	Non-frozen stored
CAA positive (n, %)	44 (16.2%)	35 (12.9%)	20 (13.1%)	16 (10.5%)	24 (15.7%)
Average CAA (pg/ml)^*^	123.9	102.8	48.8	49.2	43.7
Median CAA (pg/ml)^*^	39.2	30.0	31.6	15.0	24.4

*Average and median CAA concentrations were based on samples that were CAA-positive under both conditions being compared: fresh and frozen urine in the full data set (N = 35), and fresh, frozen and non-frozen stored urine tested in the sub-set (N = 15).

**Table 2:** Agreement and correlation between CAA results in urine samples under different conditions.

	Fresh *versus* frozen	Fresh *versus* non-frozen stored	Frozen *versus* non-frozen stored
N	272	153	153
Cohen’s kappa (95% CI)	0.87 (0.78-0.95)	0.79 (0.65-0.93)	0.71 (0.55-0.88)
McNemar p-value	0.004	0.29	0.02
Bland-Altman, mean bias (95% limits of agreement)	1.18 (0.41-3.44)	0.91 (0.30-2.77)	0.86 (0.33-2.22)
Spearman’s rho (p-value)	0.93 (<0.0001) N=35	0.87 (<0.0001) N=18	0.86 (<0.0001) N=15
Wilcoxon Z (p-value)	-1.95 (0.051)	-0.317 (0.751)	-0.848 (0.397)

**Figure 2:**
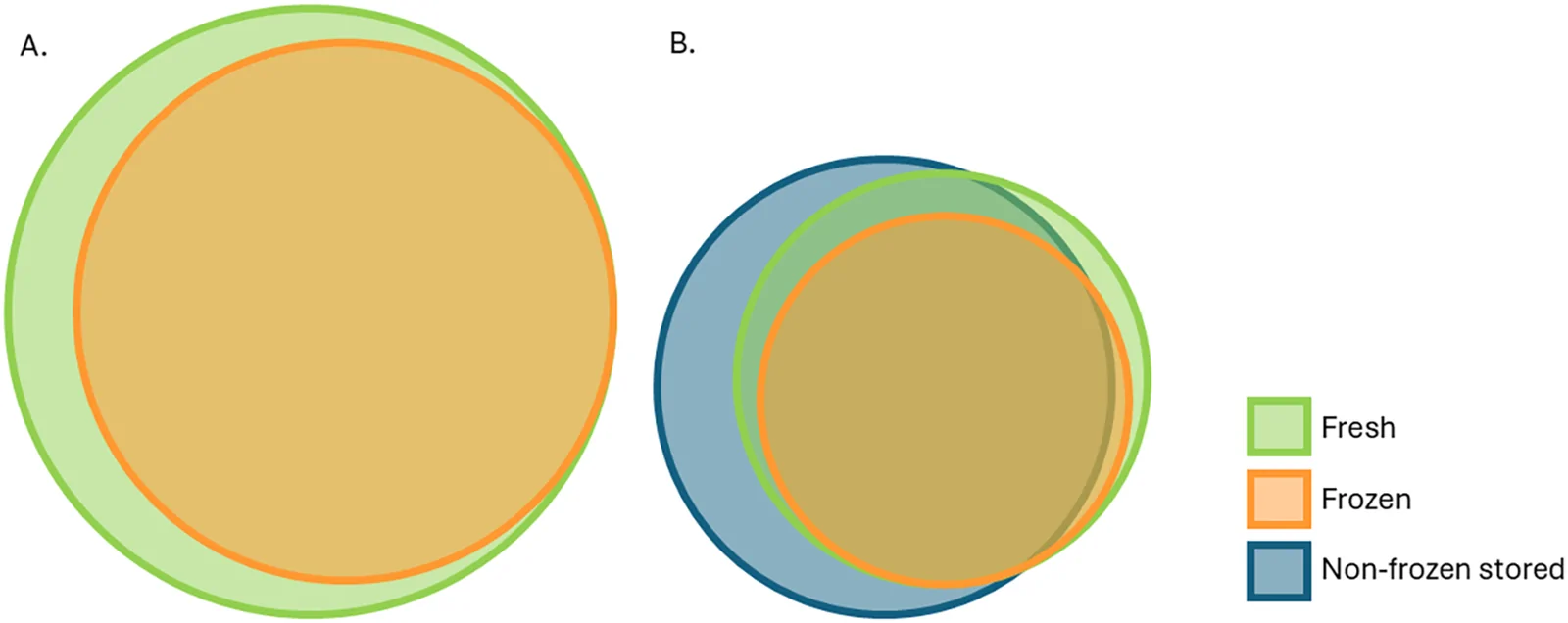
Proportional Venn diagram of CAA-positives in **(A)** fresh versus frozen urine samples (N = 272, left) and **(B)** fresh versus frozen versus non-frozen stored urine samples (N = 153, right).

**Figure 3:**
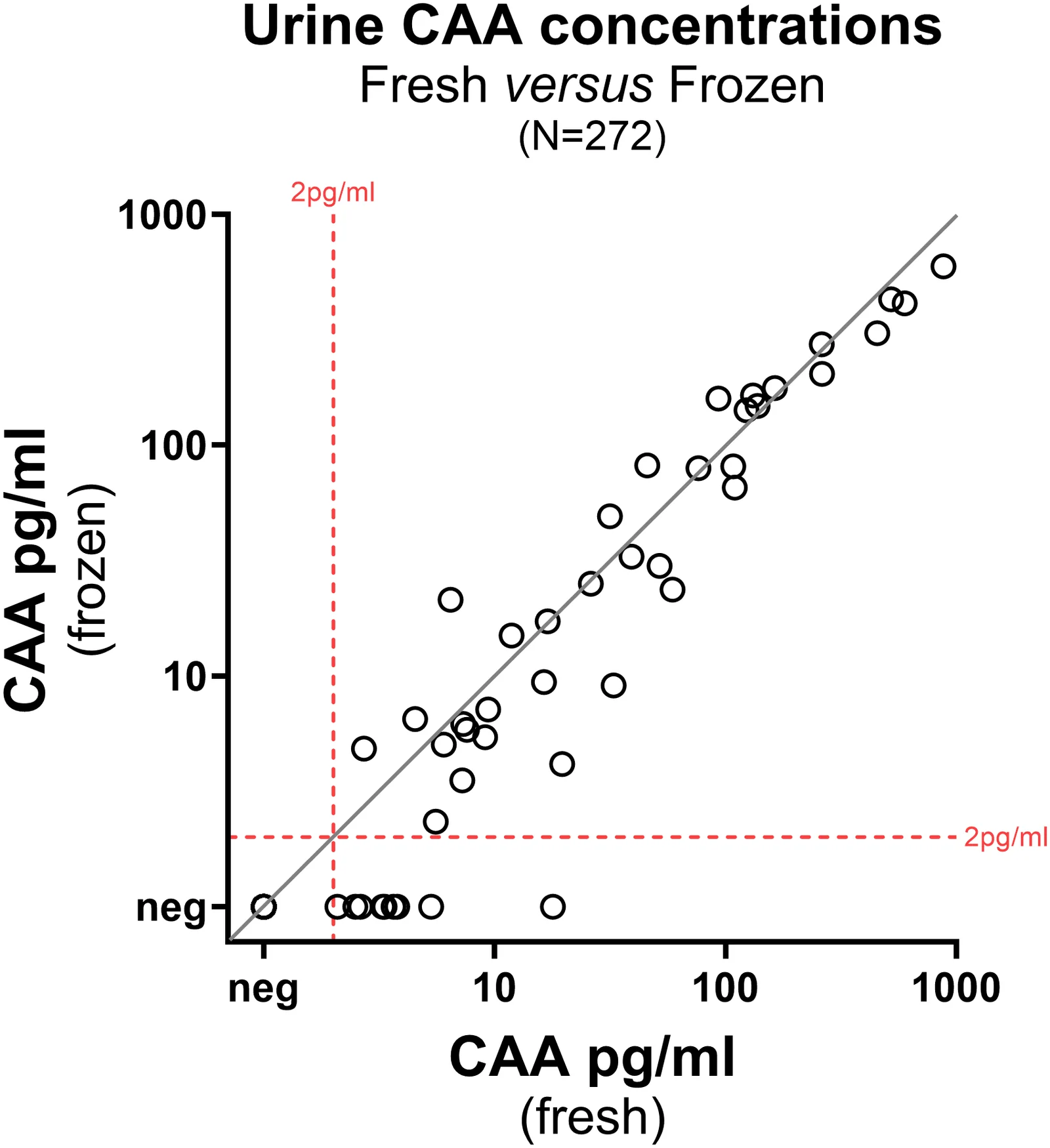
Correlation between individual CAA concentrations in fresh versus frozen urine samples (N = 272, of which 35 were double positive and 228 were double negative). CAA concentrations were determined by the UCAA*hT*417 dry format with a cut-off of 2 pg/ml (red dashed line).

For a sub-set of urine samples (N = 153), an additional aliquot was available for non-frozen storage prior to testing (**Tables 1**, **2**). The proportional Venn-diagram showed substantial overlap in CAA-positive samples between conditions (**Figure 2B**). Non-frozen stored samples showed the highest number of CAA positives (N = 24), of which 18 and 15 were also positive in the corresponding fresh and frozen samples, respectively. Compared with fresh samples, 2 samples changed from positive to negative after non-frozen storage, while 6 changed from negative to positive; this difference was not statistically significant (McNemar’s test, p=0.29; **Table 2**; **Supplementary Table 2**). CAA concentrations were comparable between both conditions (Wilcoxon signed-rank test, p=0.751; **Table 2**). In contrast, the difference in discordant pairs between non-frozen stored and frozen samples was statistically significant with 1 sample changing from positive to negative after non-frozen storage and 9 changed from negative to positive (McNemar’s test, p=0.02; **Table 2**; **Supplementary Table 2**). CAA concentrations did not differ significantly between both conditions (Wilcoxon signed-rank test, p=0.397; **Table 2**). Strong correlations were observed between quantitative CAA concentrations across all storage conditions (**Table 2**; **Figure 4**). There was no statistically significant systematic bias in CAA concentrations between fresh, frozen and non-frozen stored samples. Both fresh and frozen samples tended to have lower CAA concentrations compared to non-frozen stored samples (mean ratio 0.91, 95% limits of agreement 0.30-2.77 and mean ratio 0.86, 95% limits of agreement 0.33-2.22, respectively, **Table 2**).

**Figure 4:**
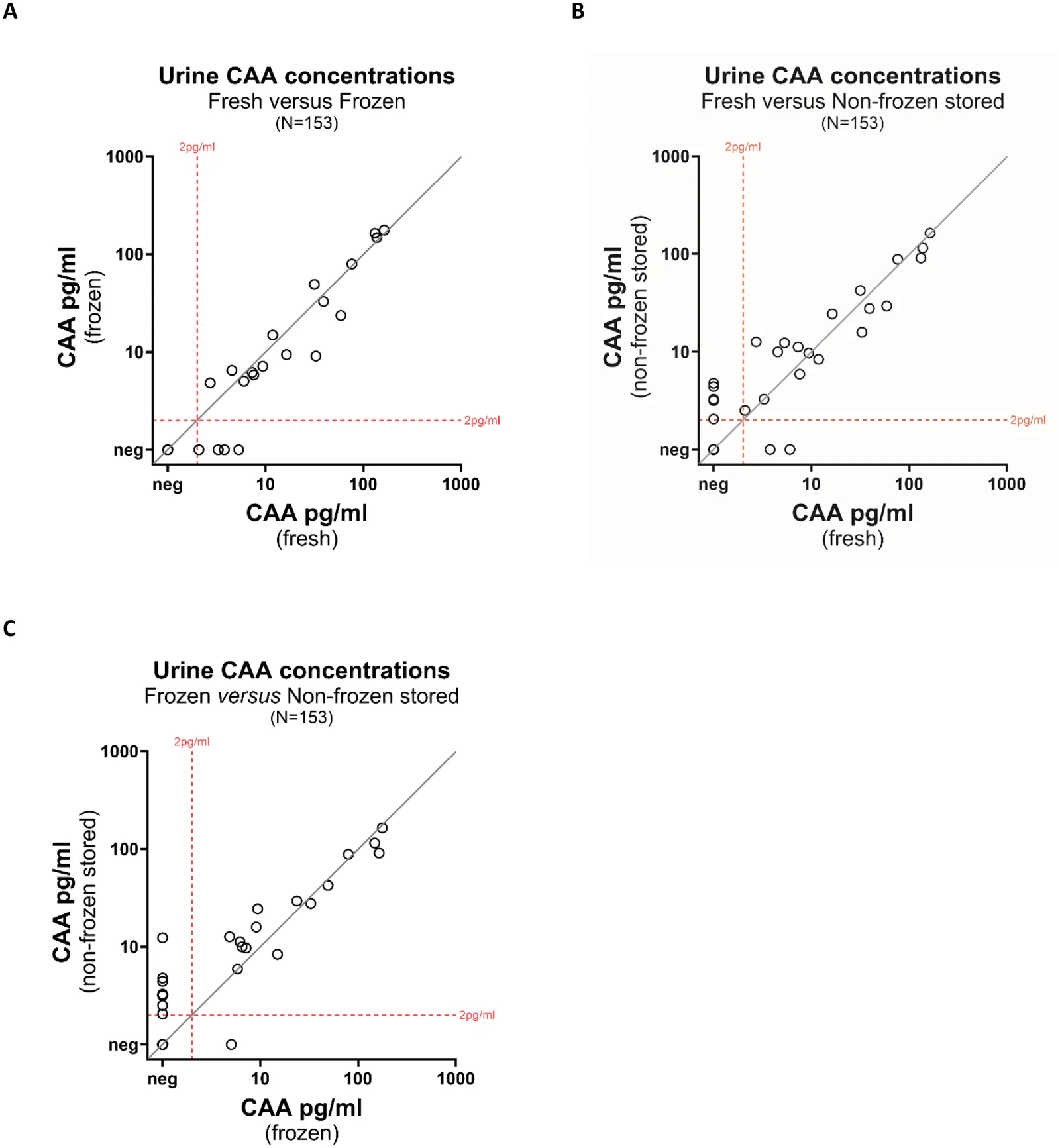
Correlation between individual CAA-concentrations in sub-set of fresh, frozen and non-frozen stored samples (N = 153). **(A)** fresh versus frozen urine samples (16 were double positive and 133 were double negative); **(B)** fresh versus non-frozen stored urine samples (18 were double positive and 127 were double negative); and **(C)** frozen versus non-frozen stored urine samples (15 were double positive and 127 were double negative). CAA concentrations were determined by the UCAA*hT*417 dry format with a cut-off of 2 pg/ml (red dashed line).

## Discussion

In this study, we evaluated the impact of freezing and non-frozen storage on the detection of circulating anodic antigen (CAA) in urine samples using the UCP-LF CAA test in a near-elimination *Schistosoma haematobium* setting on Pemba, Tanzania. Overall agreement in CAA positivity between storage conditions was high, and quantitative CAA concentrations showed good agreement. Freezing was associated with a small but statistically significant reduction in CAA positivity, while CAA concentrations showed a non-significant trend towards lower values after freezing. This reduction was observed primarily among samples with CAA concentrations close to the assay cut-off of 2 pg/ml, suggesting that analytical variability around the detection threshold may have contributed to the observed differences. Overall, our findings suggest that while CAA is generally stable and suitable for retrospective or centralized testing, freezing may lead to a limited loss of detectable antigen and should be considered when interpreting results in low-intensity settings.

Previous studies have reported stable CAA levels in urine under various storage conditions, including freezing (Corstjens et al., 2014; Corstjens et al., 2015; Corstjens et al., 2020), consistent with our current findings and previous unpublished observations from LUMC. Similarly, we did not observe a significant different in CAA positivity or concentration between fresh and non-frozen stored samples. However, this comparison cannot isolate the effect of storage from potential effects related to transport, timing of analysis, and laboratory conditions, as non-frozen samples were transported to the Netherlands and analyzed at LUMC, whereas fresh and frozen samples were analyzed on Pemba. Nevertheless, the comparable results observed across fresh and non-frozen stored samples indicate that CAA detection was maintained across this field-to-laboratory workflow. This is relevant to settings where immediate testing or freezing is not feasible and samples need to be stored and transported for centralized testing. In our study, non-frozen samples were stored at 4 °C before transport under cooled conditions using ice packs and were tested approximately 5 weeks after collection. The findings therefore demonstrate the feasibility of this specific storage and transport workflow, rather than establishing that non-frozen storage alone has no effect on CAA stability. Standardized storage and transport procedures remain important to preserve sample integrity, particularly during prolonged non-frozen storage, when bacterial or fungal growth could potentially affect CAA detection.

The non-frozen storage condition was introduced approximately half-way through the study and was assessed in a pragmatic subset for which sufficient urine was available. Although selection was not based on participant characteristics or diagnostic outcomes, selection based on available urine volume could potentially have introduced bias. In addition, this study was designed to evaluate different storage conditions rather than storage duration. Fresh samples were tested within 4 hours after collection, frozen samples were stored at -20 °C for 5–7 days before testing, and non-frozen stored samples were tested approximately 5 weeks after collection. These intervals were determined by practical and logistical circumstances rather than prospectively assigned storage periods. Therefore, the effects of storage condition and storage duration cannot be separated, and no conclusions can be drawn regarding the effect of storage duration itself. Further studies are needed to assess longer-term storage at non-frozen temperatures, prolonged frozen storage, and repeated freeze-thaw cycles.

In conclusion, the storage conditions evaluated in this study had a limited overall effect on CAA detection and quantification. Freezing for 5–7 days was associated with a small reduction in positivity, primarily among samples with CAA concentrations close to the assay cut-off, while non-frozen stored samples showed generally comparable results to freshly tested samples. These findings suggest that urine storage strategies may be adapted to facilitate delayed centralized CAA testing, while further evaluation is needed for longer storage periods and repeated freeze-thaw cycles.

## Data Availability

The datasets presented in this study can be found in online repositories. The names of the repository/repositories and accession number(s) can be found in the article/**Supplementary Material**.

## Supplementary material

Supplementary Table 1 Agreement between UCP-LF CAA results in fresh and frozen urine samples (N = 272).

Supplementary Table 2 Full dataset including CAA levels for fresh, frozen and non-frozen stored urine samples.
