# Supplementary material for: Effect of urine storage conditions on circulating anodic antigen detection using the UCP-LF CAA test
Source: Front Parasitol. 2026 Oct 5;5:1894516. doi: 10.3389/fpara.2026.1894516 (PMC13677902; doi:10.3389/fpara.2026.1894516)
Supplement: Supplementary file 1 [file Table1.docx]

**Supplementary Table 1**. Agreement between UCP-LF CAA results in fresh and frozen urine samples (N=272).

|  | | **Fresh** | | |
| --- | --- | --- | --- | --- |
|  |  | Positive | Negative | Total |
| **Frozen** | Positive | 35 | 0 | 35 |
|  | Negative | 9 | 228 | 237 |
|  | Total | 44 | 228 | 272 |

**Supplementary Table 2**. Agreement between UCP-LF CAA results in a sub-set of fresh, frozen, and non-frozen stored urine samples (N=153).

|  | | **Fresh** | | |
| --- | --- | --- | --- | --- |
|  |  | Positive | Negative | Total |
| **Frozen** | Positive | 16 | 0 | 16 |
|  | Negative | 4 | 133 | 137 |
|  | Total | 20 | 133 | 153 |
|  | | **Fresh** | | |
|  |  | Positive | Negative | Total |
| **Stored** | Positive | 18 | 6 | 24 |
|  | Negative | 2 | 127 | 129 |
|  | Total | 20 | 133 | 153 |
|  |  | **Frozen** | | |
|  |  | Positive | Negative | Total |
| **Stored** | Positive | 15 | 9 | 24 |
|  | Negative | 1 | 128 | 129 |
|  | Total | 16 | 137 | 153 |
